# Supplementary material for: Spatiotemporal organization of branched microtubule networks
Source: eLife. 2019 May 8;8:e43890. doi: 10.7554/eLife.43890 (PMC6519983; doi:10.7554/eLife.43890)
Supplement: Transparent reporting form [file elife-43890-transrepform.pdf]

eLIFE

1st Floor
24 Hills Road
Cambridge CB2 1JP, UK

P 01223 855340
W elifesciences.org
T @elife

eLife's transparent reporting form

We encourage authors to provide detailed information *within their submission* to facilitate the interpretation and replication of experiments. Authors can upload supporting documentation to indicate the use of appropriate reporting guidelines for health-related research (see EQUATOR Network), life science research (see the BioSharing Information Resource), or the ARRIVE guidelines for reporting work involving animal research. Where applicable, authors should refer to any relevant reporting standards documents in this form.

If you have any questions, please consult our Journal Policies and/or contact us: editorial@elifesciences.org.

Sample-size estimation

- You should state whether an appropriate sample size was computed when the study was being designed
- You should state the statistical method of sample size computation and any required assumptions
- If no explicit power analysis was used, you should describe how you decided what sample (replicate) size (number) to use

Please outline where this information can be found within the submission (e.g., sections or figure legends), or explain why this information doesn't apply to your submission:

Number of measurements (sample size) performed for each measurement are stated in each individual figure legend for all experiments, simulations and measurements made.

No sample size calculation were performed and sample sizes were chosen with the following rationale for individual experiments.

For complete tracking and analysis of branched networks to obtain parameters, tens of branched networks were imaged for 5-15 minutes for each independent egg extract preparation.The reaction was repeated several times to acquire 10-20 branched network for each Xenopus egg extract preparation. All networks were tracked and all data was pooled and reported in Figure 1- supplement 2. Upon analyses, the results from each branched networks were in agreement with others and gave us consistent measurements. All data was pooled and reported. The experiment was repeated more than two times with independent Xenopus egg extract preparation.

For measurement of spatial nucleation profile in Figure 2A-B, Figure 3 and related supplemental figures, the reactions in Xenopus egg extracts were imaged immediately after preparation. This gave us 5-10 naked mother [illegible]

Replicates

- You should report how often each experiment was performed
- You should include a definition of biological versus technical replication
- The data obtained should be provided and sufficient information should be provided to indicate the number of independent biological and/or technical replicates
- If you encountered any outliers, you should describe how these were handled
- Criteria for exclusion/inclusion of data should be clearly stated
- High-throughput sequence data should be uploaded before submission, with a private link for reviewers provided (these are available from both GEO and ArrayExpress)

Please outline where this information can be found within the submission (e.g., sections or figure legends), or explain why this information doesn't apply to your submission:

The information on sample size and replicates are provided for each individual experiment, simulation or measurement made in the respective figure legends. No data or outliers were excluded from the analyses.

eLife Sciences Publications, Ltd is a limited liability non-profit non-stock corporation incorporated in the State of Delaware, USA, with company number 5030732, and is registered in the UK with company number FC030576 and branch number BR015634 at the address 1st Floor, 24 Hills Road, Cambridge CB2 1JP | August 2014

Statistical reporting

- Statistical analysis methods should be described and justified
- Raw data should be presented in figures whenever informative to do so (typically when N per group is less than 10)
- For each experiment, you should identify the statistical tests used, exact values of N, definitions of center, methods of multiple test correction, and dispersion and precision measures (e.g., mean, median, SD, SEM, confidence intervals; and, for the major substantive results, a measure of effect size (e.g., Pearson's r, Cohen's d)
- Report exact p-values wherever possible alongside the summary statistics and 95% confidence intervals. These should be reported for all key questions and not only when the p-value is less than 0.05.

Please outline where this information can be found within the submission (e.g., sections or figure legends), or explain why this information doesn't apply to your submission:

In all our measurements, the number of measurements were greater than or equal to 10 and therefore their probability distributions, or mean and SD, are displayed. For Figure1- supplement 2C, individual time series (n=11) are displayed.

Statistical methods are reported and the values displayed (SD, mean, median, confidence intervals) are described clearly in the figure legends for each panel displayed.

(For large datasets, or papers with a very large number of statistical tests, you may upload a single table file with tests, Ns, etc., with reference to sections in the manuscript.)

Group allocation

- Indicate how samples were allocated into experimental groups (in the case of clinical studies, please specify allocation to treatment method); if randomization was used, please also state if restricted randomization was applied
- Indicate if masking was used during group allocation, data collection and/or data analysis

Please outline where this information can be found within the submission (e.g., sections or figure legends), or explain why this information doesn't apply to your submission:

Samples were not required to be allocated into experimental groups. Various protein concentrations in Xenopus egg extracts were changed and identical experiments were performed after changes in the protein concentrations. This information therefore does not apply to this submission.

Additional data files ("source data")

- We encourage you to upload relevant additional data files, such as numerical data that are represented as a graph in a figure, or as a summary table
- Where provided, these should be in the most useful format, and they can be uploaded as "Source data" files linked to a main figure or table
- Include model definition files including the full list of parameters used
- Include code used for data analysis (e.g., R, MatLab)
- Avoid stating that data files are "available upon request"

Please indicate the figures or tables for which source data files have been provided:

All model definitions and list of parameters used are described clearly in the main text and supplemental figures in table format. All code (MATLAB) used for data analysis has been provided as an additional file with the manuscript as "MATLAB software" zip folder. All data generated in this study was pooled (no data was left out) and reported as probability distributions in the figures. Source data is therefore not needed and was not provided.
